# Unsupervised Manifold Learning Using High-Order Morphological Brain Networks Derived From T1-w MRI for Autism Diagnosis

**DOI:** 10.3389/fninf.2018.00070

**Published:** 2018-10-26

**Authors:** Mayssa Soussia, Islem Rekik

**Affiliations:** ^1^CVIP Group, BASIRA Lab, School of Science and Engineering, Computing, University of Dundee, Dundee, United Kingdom; ^2^Department of Electrical Engineering, The National Engineering School of Tunis, Tunis, Tunisia

**Keywords:** morphological brain network, high-order brain connectivity, multi-kernel learning, classification, diagnosis, Autism Spectrum Disorder, hierarchical ensemble classifier, morphological connectional biomarkers

## Abstract

Brain disorders, such as Autism Spectrum Disorder (ASD), alter brain functional (from fMRI) and structural (from diffusion MRI) connectivities at multiple levels and in varying degrees. While unraveling such alterations have been the focus of a large number of studies, *morphological brain connectivity* has been out of the research scope. In particular, shape-to-shape relationships across brain regions of interest (ROIs) were rarely investigated. As such, the use of networks based on morphological brain data in neurological disorder diagnosis, while leveraging the advent of machine learning, could complement our knowledge on brain wiring alterations in unprecedented ways. In this paper, we use conventional T1-weighted MRI to define morphological brain networks (MBNs), each quantifying shape relationship between different cortical regions for a specific cortical attribute at both *low-order* and *high-order* levels. While typical brain connectomes investigate the relationship between two ROIs, we propose high-order MBN which better captures brain complex interactions by modeling the morphological relationship between pairs of ROIs. For ASD identification, we present a connectomic manifold learning framework, which learns multiple kernels to estimate a similarity measure between ASD and normal controls (NC) connectional features, to perform dimensionality reduction for clustering ASD and NC subjects. We benchmark our ASD identification method against both supervised and unsupervised state-of-the-art methods, while depicting the most discriminative high- and low-order relationships between morphological regions in the left and right hemispheres.

## 1. Introduction

Autism Spectrum Disorder (ASD) is a neurodevelopmental disorder characterized by varied impairments in cognitive function, including difficulties with social communication and interaction, language, and restricted, repetitive behaviors (Lord et al., 2000; Landa, 2008). Recent technological and methodological advances in neuroimaging tools have largely aided in understanding how ASD alters the brain, in particular on a connectional level where the connectivity between brain regions of interest (ROIs) is estimated. However, due to its heterogeneity (Lenroot and Yeung, 2013; Masi et al., 2017), depicting the core connectional patterns of ASD disorder is a challenging task. The two most commonly used representations of brain connectivity in the neuroscience literature are functional and structural networks, estimated from functional and diffusion-weighted Magnetic Resonance Imaging (MRI), respectively.

On a functional connectivity level, a lot of effort has been put to discover the connectional fingerprint of ASD disorder across its wide spectrum. Using the technique of graph theory (Bullmore and Bassett, 2011; Rudie et al., 2013) showed that differences in functional connectivity (FC) of ASD subjects are associated with reductions in modularity and shorter characteristic path lengths while the structural networks displayed lower levels of white matter. Additionally, Sato et al. (2016) identified a set of spatially distributed regions that were disrupted in their modularity compared to controls based on a clustering entropy with graph modularity analysis on a resting state fMRI data. Tsiaras et al. (2011) used a well-established graph measures which served as features in classifying controls and young adults with ASD. Pillai et al. (2018) used the electroencephalography (EEG) and a movement-based paradigm to examine the FC changes in ASD children while performing specific tasks. Anderson et al. (2011) characterized a whole-brain functional connectivity abnormalities in a data-driven fashion to identify the regions showing greatest differences between individual subjects with autism and developing controls. A similar work (Nielsen et al., 2013) used also the whole-brain FC across sites to determine the most informative patterns for predicting autism but compared to a single site results, it exhibited poorer accuracy. Furthermore, by generating the connectivity maps based on Granger causality, Pollonini et al. (2010) indicated that functional patterns can represent a valuable tool to separate between autistic and normal groups. In addition to this, multiple studies have attempted to explore the merits of dynamic connectivity features derived from resting state fMRI in discriminating childhood autism (Price et al., 2014; Zhu et al., 2016).

On a structural connectivity level (Sparks et al., 2002), examined morphometric features of a large samples of children with ASD and control groups to explore the specific neuroanatomic substrates associated with this disorder. (Ecker et al., 2010) approached a multiparameter classification based on volumetric and geometric features to characterize the structural patterns implicated in autistic adults. In another study (Ecker et al., 2009), the predictive values of gray and white matter was investigated using two different classifiers to compare the results. Moreover, Ingalhalikar et al. (2010) learned an abnormality classifier on structural features derived from Diffusion Tensor Imaging (DTI) to quantify the degree of pathology among a population of patients and normal controls. Additionally, a lot of studies combined the functional and structural connectivity networks for the aim of providing more biomarkers for ASD identification (Sahyoun et al., 2010; Stigler et al., 2011; Rudie et al., 2013).

Despite the wealth of research relying on the functional and structural connectivity networks for brain disease diagnosis, these brain connectional representations have a few limitations. For instance, pairwise FC strength among brain regions can be spurious and noisy due to the low signal-to-noise ratio induced by non-neural noise. Moreover, fMRI measures during the scans can be sensitive to a group of factors such as head motion and physiological artifacts related to respiration and cardiac rhythm (Buckner et al., 2013). On the other hand, fiber tractography methods can produce largely variable and somewhat biased structural brain networks (Jbabdi and Johansen-Berg, 2011). Indeed, a recent study (Petrov et al., 2017) evaluated 35 methods to generate structural connectomes and showed that how variations in diffusion MRI pre-processing steps affect network reliability and its ability to classify subjects remains opaque. With the exception of the high-resolution diffusion imaging (HARDI) and diffusion orientation distribution functions (ODFs) fiber representation, which are memory and time consuming to process, commonly used diffusion tensor imaging (DTI) can lead to a loss of information in fiber pathways as it assumes a single predominant orientation of fibers in the brain (Lanyon, 2012). To circumvent the limitations of these modalities, we propose an alternative brain network representation: a morphological brain network (MBN) solely constructed from structural T1-w MRI. The main idea is to build a network based on the morphology of the cortical surface, where each network is associated with a unique cortical attribute such as sulcal depth or cortical thickness. Our conventional MBN is defined at a low-order level, where the dissimilarity in shape between two brain regions is quantified. However, recent functional MRI-based studies have shown that ASD not only affects the relationship between two ROIs, but also pairs of ROIs captured by high-order functional brain connectivity (Zhao et al., 2018; Zhou et al., 2018). Other works investigated the relationship between brain network views using the multiplex architecture for dementia state identification (Lisowska et al., 2017; Lisowska and Rekik, 2018; Mahjoub et al., 2018). Inspired by these works which represent the brain as a complex multi-order connectional system, we introduce high-order morphological brain networks, which capture the relationship between cortical attributes across pairs of ROIs, for autism identification.

We also note that all aforementioned studies adopted supervised techniques on human connectome for ASD/NC classification (Ecker et al., 2009, 2010; Ingalhalikar et al., 2010; Zhao et al., 2018; Zhou et al., 2018). However, while the majority of supervised machine-learning techniques are somewhat limited in terms of scalability as they require reliable and accurate labeling of medical data, unsupervised learning techniques can provide decision support for early intervention, help develop data-driven guidelines for care plan management, and help group patients by similar non-semantic features (i.e., latent representation of brain disorder group or subgroup), to enable comparative effectiveness research (e.g., of medications) (Wang et al., 2014). From a connectomic perspective, very few studies applied unsupervised learning methods for brain disease applications (Brown and Hamarneh, 2016). For instance, (Gao et al., 2015) computed spectral graph clustering to identify significant connectome modules for different brain disorder groups [Alzheimer's disease (AD) and Significant Memory Concern (SMC)]. Another work (Chen et al., 2015) used a multi-view spectral clustering to group functional and structural brain networks of traumatic brain injury (TBI) patients. On the other hand, in distinguishing between autistic and healthy brains, we identified only one paper (Sato et al., 2016) that adopted an unsupervised learning where the author used a fuzzy spectral clustering combined with entropy and graph modularity analysis. However, spectral clustering might fail to successfully group datasets that contain different scales of size and density in their structures (Nadler and Galun, 2006).

To overcome the previous limitations, we propose a high-order morphological connectomic manifold learning framework for ASD identification inspired by a novel unsupervised data clustering method called single-cell interpretation via multikernel learning (SIMLR) (Wang et al., 2017). Our choice for leveraging this algorithm is motivated by: (1) SIMLR can learn a similarity matrix from high-order networks by combining multiple kernels which provides a better fit to the inherent statistical distribution of the high-order data, (2) it is scalable and separates subpopulations more accurately than conventional methods (e.g., PCA Abdi and Williams, 2010 or t-SNE Maaten and Hinton, 2008), and (3) it improves weak similarities between samples through graph diffusion (Yang and Leskovec, 2010).

This paper further extends our seminal work (Soussia and Rekik, 2017) by: (1) evaluating the proposed approach on a larger dataset, (2) comparing against more advanced supervised ensemble learning approaches to show the outperformance of our unsupervised learning framework using multi-order brain networks. More importantly, we identify the key low-order and high-order morphological connectional features that distinguish between ASD and NC subjects for each cortical hemisphere.

## 2. Methods

**Figure 1:**
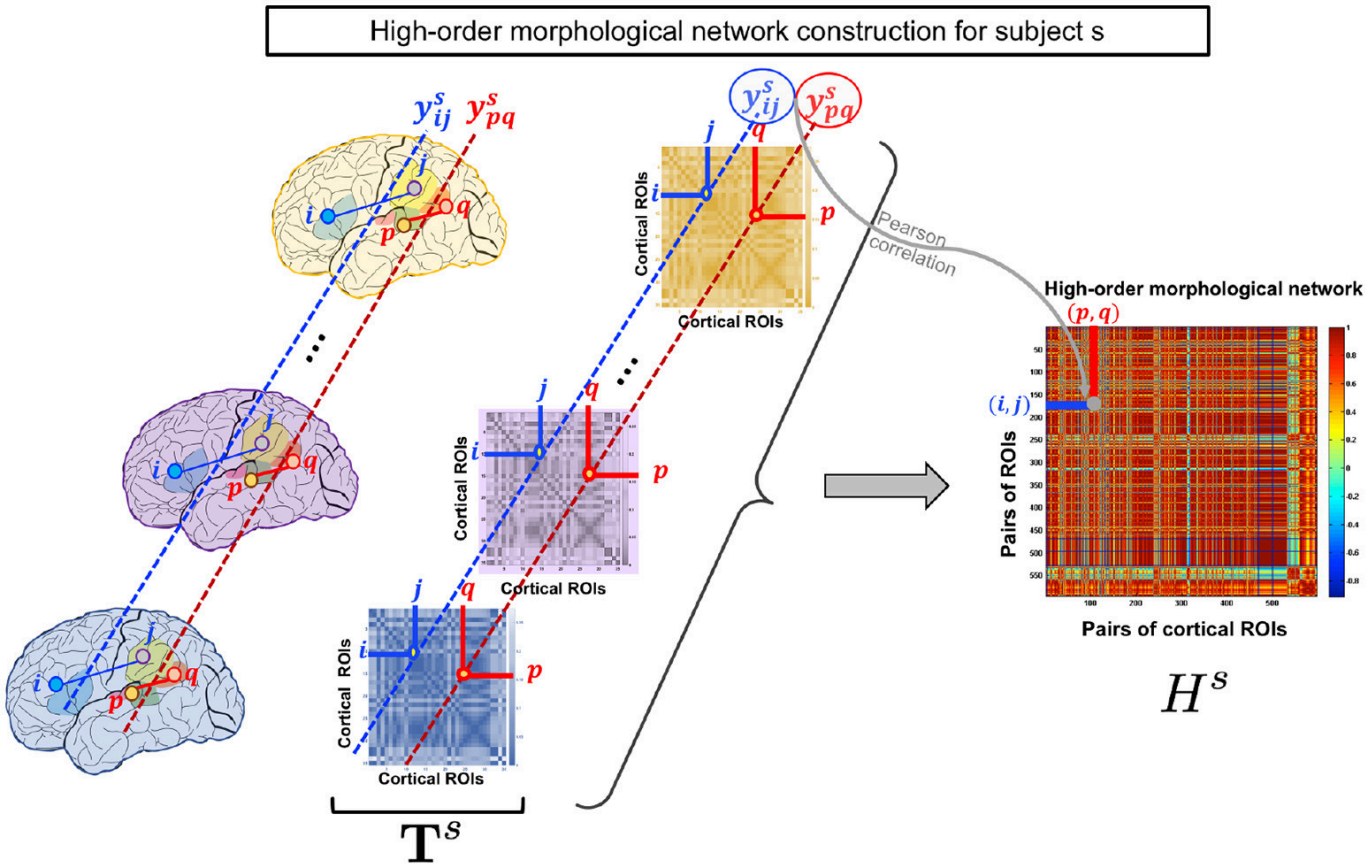
High-order morphological network construction for subject *s*. High-order morphological network construction using multiple brain networks, each measuring a unique cortical attribute (e.g., thickness) on the cortical surface. These are stacked together to form a morphological brain tensor $T^{s}$ for subject *s*.

**Figure 2:**
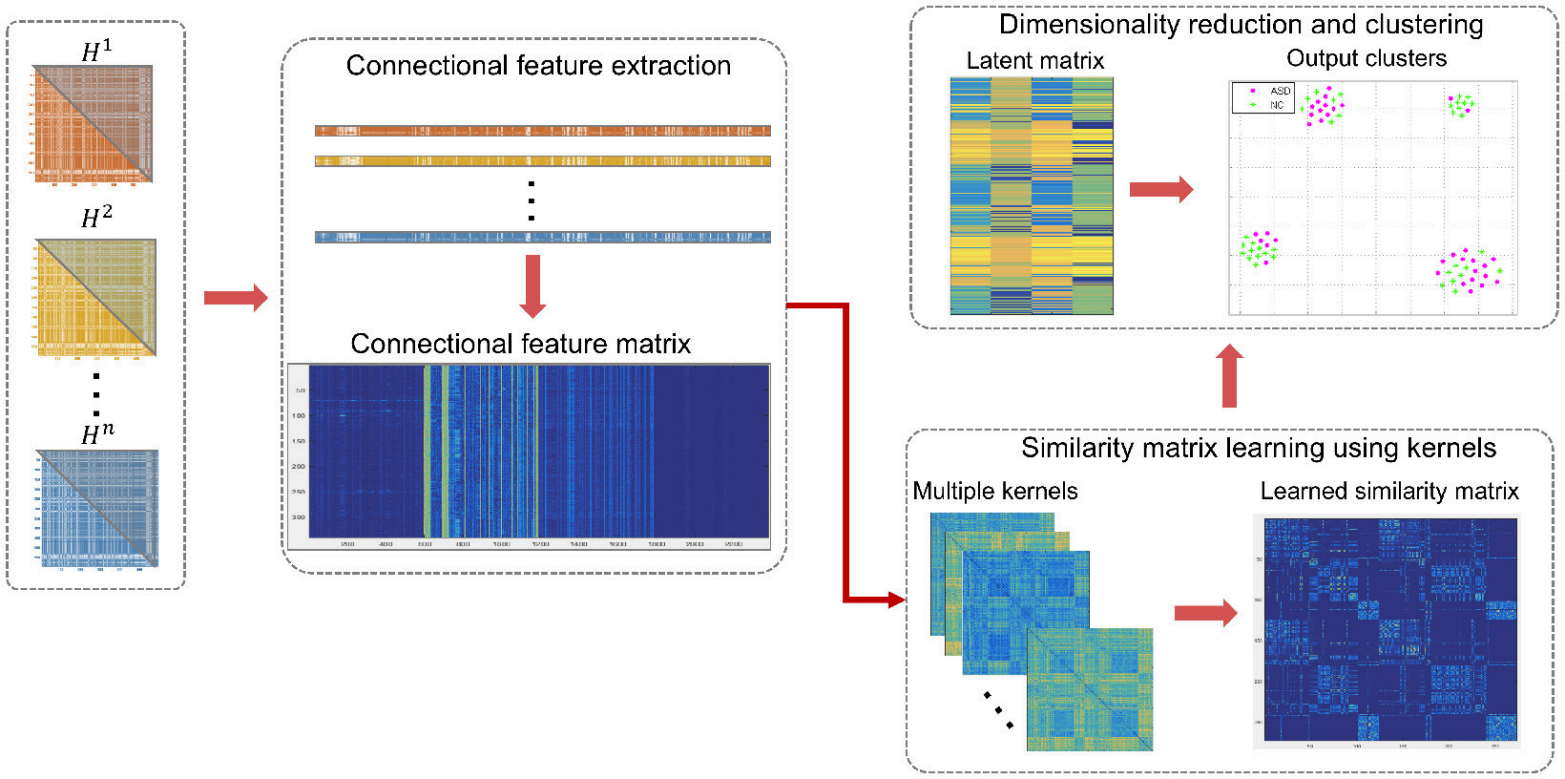
Illustration of the proposed high-order connectomic manifold learning for autistic brain state identification. Given the high-order feature matrix of all subjects, we used SIMLR (Wang et al., 2017) to learn proper weights for multiple kernels, which measure different distances between subjects. Next, we use the learned kernels to construct a symmetric similarity matrix **S** between subjects. SIMLR imposes a low-rank constraint on **S** such that different populations of the input data will be embedded into independent block-diagonal structure that clusters similar samples. This outputs a latent data representation in a low-dimensional space, which is inputted to a clustering algorithm. Each point in the 2D scatter plot represents an ASD or NC subject, and the corresponding colors represent the true labels in each cluster.

In this section, we present the high-order connectomic manifold learning for ASD identification using multiple kernels based on SIMLR technique introduced in Wang et al. (2017). We denote tensors by boldface Euler script letters, e.g., X. Matrices are denoted by boldface capital letters, e.g., **X**, and scalars are denoted by lowercase letters, e.g., *x*. For easy reference and enhancing the readability, we have summarized the major mathematical notations in Table 1. Figure 1 displays the key steps for constructing low-order and high-order morphological network from a set of *n*_*v*_ brain network views. Figures 1, 2 illustrate the proposed pipeline for ASD/NC identification which consists of three major steps: (1) connectional morphological feature extraction, (2) subject-to-subject similarity matrix learning using SIMLR, and (3) dimensionality reduction and clustering of our features.

**Table 1:** Major mathematical notations used in this paper.

**Mathematical notation**	**Definition**
$T^{s}$	brain tensor of subject *s* in ${\mathbb{R}}^{n_{r}\times n_{r}\times n_{v}}$
**X**^*k*^	brain network in ${\mathbb{R}}^{n_{r}\times n_{r}}$ denoting the *k*-th frontal-view of tensor T
${\text{y}}_{ij}^{s}$	vector of subject *s* including connectivity weights between the *i*-th and *j*-th ROIs across all views
**H**^*s*^	high-order morphological brain network for subject *s*
**h**_*s*_	high-order feature vector extracted from the upper triangular part of **H**^*s*^
**K**_*l*_	*l*-th learning kernel in ℝ^*n*×*n*^
*n*	number of subjects
**S**	similarity matrix in ℝ^*n*×*n*^ for connectomic manifold learning
**L**	latent matrix in ℝ^*n*×*c*^
*c*	number of clusters
*m*	number of kernels
**w**	weighting vector of the kernels in ℝ^*m*^
**I**_*n*_	identity matrix in ℝ^*n*×*n*^

### 2.1. Low-order morphological network construction (LON)

In line with the works of Lisowska et al. (2017), Lisowska and Rekik (2018), and Mahjoub et al. (2018) which laid the foundation for defining multi-view brain networks from cortical morphology, we use T1-w MRI to define our low-order networks as follows. For each subject *s*, we construct a brain tensor $T^{s}$ of size ${\mathbb{R}}^{n_{r}\times n_{r}\times n_{v}}$ for each cortical hemisphere, where *n*_*r*_ is the number of cortical regions of interest (ROIs) and *n*_*v*_ is the number of the tensor frontal views. Basically, for each cortical attribute (e.g., thickness), we construct a morphological brain network that constitutes a frontal view in $T^{s}$. Let $x_{i}^{k}$ and $x_{j}^{k}$ denote the mean of a cortical attribute of the *i*-th ROI and the *j*-th ROI in the *k*-th frontal view respectively. We then compute the absolute difference between $x_{i}^{k}$ and $x_{j}^{k}$ which depicts the connectivity weight between ROIs *i* and *j*. An element in the *i*-th row and *j*-th column of the *k*-th frontal view **X**^*k*^ is defined as: ${\text{X}}_{ij}^{k}=|x_{i}^{k}-x_{j}^{k}|$.

### 2.2. High-order morphological network construction (HON)

As the low-order network is unable to reveal the intrinsic similarities between more than a pair of ROIs, we propose to construct a high-order morphological network based on Pearson correlation to detect more complex interaction patterns between multiple brain regions. In addition to maintaining the pairwise relationship between ROIs in the same morphological view, the morphological HON underlines the relationship between ROIs across different views. Let ${\text{y}}_{ij}^{s}$ denote the vector of subject *s* including connectivity weights between the *i*-th and *j*-th ROIs across all views. Each row in the high-order network **H**^*s*^ represents a pair of ROIs (*i, j*) and each column denotes a pair of ROIs (*p, q*). For a subject *s*, an element in *H*^*s*^ is defined using the Pearson's correlation coefficient as ${\text{H}}_{ij,pq}^{s}=corr\left({\text{y}}_{ij}^{s},{\text{y}}_{pq}^{s}\right)$. We note that the entries ${\text{H}}_{ij,pq}^{s}$ of the HON matrix indicate the connectivity strength between ROIS (*i, j*) and (*p, q*). Thus, it underlines the higher order relationship between multiple ROIs (Figure 1).

### 2.3. Feature extraction

For each subject, features are extracted in a naive way. Due to their symmetry, we concatenate the upper triangle elements of the HON matrix for subject *s* into a long feature vector **h**^*s*^. As for the LON, we simply concatenate the extracted feature vector from each network view. The weights on the diagonal are set to zero to avoid self-connectedness.

### 2.4. Unsupervised manifold learning using high-order morphological brain networks

In this section, we briefly present the framework introduced in Wang et al. (2017) and how we extended it to our aim. The main idea of SIMLR is to learn a pairwise similarity matrix of size *n* × *n* from an input matrix of size *n* × *d* where *n* is the number of subjects and *d* is the dimension of their associated feature vectors. This allows to learn the connectomic manifold where all HON features {**h**^1^, …, **h**^*n*^} are nested. Instead of using one predefined distance metric which may fail to capture the nonlinear relationship in the data, we use multiple Gaussian kernels with learned weights to better explore in depth the similarity patterns among ASD and NC HONs. In other words, adopting multiple kernels allows to better fit the true underlying statistical distribution of the input matrix of high-order features. Additionally, constraints are imposed on kernel weights to avoid a single kernel selection (Wang et al., 2017). The Gaussian kernel is expressed as follows: $\text{K}\left({\text{h}}^{i},{\text{h}}^{j}\right)=\frac{1}{\epsilon_{ij}\sqrt{2\pi }}e^{\left(-\frac{|{\text{h}}^{i}-{\text{h}}^{j}{|}^{2}}{2\epsilon_{ij}^{2}}\right)}$, where **h**^*i*^ and **h**^*j*^ denote the feature vectors of the *i*-th and *j*-th subjects respectively and ϵ_*ij*_ is defined as: ϵ_*ij*_ = σ(μ_*i*_ + μ_*j*_)/2, where σ is a tuning parameter and $\mu_{i}=\frac{\underset{l\in KNN\left({\text{h}}^{i}\right)}{\sum }|{\text{h}}^{i}-{\text{h}}^{j}|}{k}$, where *KNN*(**h**^*i*^) represents the top *k* neighboring subjects of subject *i*. The computed kernels are then averaged to further learn the similarity matrix **S** through an optimization framework formulated as follows:

(1) $$\begin{array}{l} \underset{{}_{S,L,w}}{\min}\sum_{i,j}-w_{l}K_{l}\left(h^{i},h^{j}\right)S_{ij}+\beta \|S{\|}_{F}^{2}+\gamma tr\left(L^{T}\left(I_{n}-S\right)L\right)\\ \;+\rho \sum_{l}w_{l}logw_{l} \end{array}$$

Subject to: $\underset{l}{\sum }w_{l}=1$, *w*_*l*_ ≥ 0, ${\text{L}}^{T}\text{L}={\text{I}}_{c}$, $\underset{j}{\sum }\;{\text{S}}_{ij}=1$, and **S**_*ij*_ ≥ 0 for all (*i, j*), where:

1. $\underset{i,j}{\sum }-w_{l}{\text{K}}_{l}\left({\text{h}}^{i},{\text{h}}^{j}\right){\text{S}}_{ij}$ refers to the relation between the similarity and the kernel distance with weights *w*_*l*_ between two subjects. The learned similarity should be small if the distance between a pair of subjects is large.
2. $\beta \|\text{S}{\|}_{F}^{2}$ denotes a regularization term that avoids over-fitting the model to the data.
3. $\gamma \text{tr}\left({\text{L}}^{T}\left({\text{I}}_{n}-\text{S}\right)\text{L}\right)$: **L** is the latent matrix of size *n* × *c* where *n* is the number of subjects and *c* is the number of clusters. The matrix (**I**_*n*_ − **S**) denotes the graph Laplacian.
4. $\rho \underset{l}{\sum }w_{l}logw_{l}$ imposes constraints on the kernel weights to avoid selection of a single kernel.

An alternating convex optimization is adopted where each variable is optimized while fixing the other variables until convergence (Wang et al., 2017). Once, the similarity matrix **S** is obtained, a dimensionality reduction is performed on **S** using t-SNE (Maaten and Hinton, 2008). In other words, the data is projected onto a lower dimension that preserves the similarity depicted in **S** resulting in an *n* × *c* latent matrix **L**. For visualization, the same algorithm is used to create an embedding of **S** in a 2D space. A K-means clustering is then applied to the latent matrix **L** to cluster similar subjects and assess the concordance with the true labels (Figure 1). It should be noted that the true labels were only used in the form of distinct colors to intuitively visualize the groups in (Figure 2).

### 2.5. Proposed supervised ensemble classification methods

Previous research showed that supervised ensemble classifier tend to be more accurate than the individual classifiers that make them up (Džeroski and Ženko, 2004; Quan et al., 2016). There are many advantages of the ensemble learning. *First*, when only a small dataset is available for training, many different hypotheses can give the same accuracy on training data. Ensemble might alleviate this problem by taking an average of these hypotheses (Dietterich, 2000). *Second*, ensemble classifier can provide a good approximation of target function when the true target function cannot be represented by any of the hypotheses (i.e., by taking a weighted sum of these hypotheses) (Dietterich, 2000; Quan et al., 2016). *Third*, by combining multiple classifiers, ensemble learning reduces the sensitivity to the shape of the training data due to its limited size, leading to a better generalization of the trained model (Quan et al., 2016). *Fourth*, ensemble classifier helps alleviate problems connected to the imperfectness of the learning algorithm used –i.e., it allows for the combination of multiple linear classifiers for classification of linearly inseparable data, while keeping the simplicity of the model instead of using highly nonlinear classifier (Quan et al., 2016). Leveraging the strengths of ensemble learning, we propose supervised ensemble classifier learning using multiple sets of paired clusters obtained in an unsupervised way and on each pair a Support Vector Machine (SVM) classifier is trained. Specifically, we propose novel boosted supervised learning techniques: (1) SIMLR-based pairing + SVM, and (2) Hierarchical Ward's linkage Clustering based pairing strategy + SVM (HWC-based pairing + SVM). Basically, we apply SIMLR (respectively HWC) on ASD samples then NC subjects separately. Our aim is to disentangle heterogeneous samples within the same group. For a given number *c* of clusters, each group of ASD and NC subjects is split into *c* subgroups. Afterwards, each ASD subgroup is paired with an NC subgroup, thereby generating *c*^2^ possible pairings of subgroups, which will be merged to create a new training subset. Next, we train an SVM classifier on each merged subgroup of ASD/NC subjects while adopting a leave-one-out (LOO) scheme. Finally, a new testing subject will be evaluated by each SVM, trained using a specific pair, thereby predicting its label (i.e., ASD or NC). We then use majority voting across all SVMs to predict the final label.

## 3. Results

### 3.1. Evaluation dataset and parameters

We evaluated the proposed clustering framework on 341 subjects (155 ASD and 186 NC) from Autism Brain Imaging Data Exchange (ABIDE I)^1^ public dataset, each with structural T1-w MR image (Mueller et al., 2005). Table 2 displays the data distribution. We used FreeSurfer (Fischl, 2012) to reconstruct both right and left cortical hemispheres for each subject from T1-w MRI. Then we parcellated each cortical hemisphere into 35 cortical regions using Desikan-Killiany Atlas. For each subject, we generated *n*_*v*_ = 4 cortical morphological networks: **X**^1^ denotes the maximum principal curvature brain view, **X**^2^ denotes the mean cortical thickness brain view, **X**^3^ denotes the mean sulcal depth brain view, and **X**^4^ denotes the mean of average curvature. For SIMLR parameters, using a nested grid search, we set the number of clusters to *c* = 4. We used *m* = 21 kernels where each kernel is determined by a set of hyperparameters (σ = 1:0.25:2.5, number of top KNN neighbors in {10, 12, 14}), where σ is the variance parameter of the Gaussian function.

**Table 2:** Table of data distribution.

	**ASD**	**NC**
M	140	155
F	15	31
Total	155	186
Mean age	16.9	16.6
Std age	6.3	6.0

*M, male; F, female; Total, total number of subjects in each group; Std, standard deviation*.

### 3.2. Evaluation, reproducibility, and comparison methods

To evaluate the performance and reproducibility of our proposed clustering framework, we adopted two different k-fold cross-validation schemes (*k* = 5 and *k* = 10), where data samples were randomly partitioned into a training set and a testing set. Next, the training samples were clustered into four groups and the performance rate was calculated based on the misclassified points in each cluster. The process was repeated 20 times and the average classification rate was reported as final result for all comparison methods. To further assess the efficiency of our method, we benchmarked it to a variety of baseline methods: supervised, unsupervised, and a combination of both (e.g., HWC-based pairing + SVM, SIMLR-based pairing + SVM). First, we compared our ASD/NC clustering with the popular supervised SVM, which learns a single hyperplane to discriminate between two groups using training connectomic features. Second, we benchmarked our method against Ward's linkage clustering (Joe and Ward, 1963), a widely used hierarchical clustering algorithm which optimizes a Euclidean objective function as a criterion for merging a pair of clusters at each step. This method was previously used for clustering high-order functional networks for Alzheimer's disease diagnosis (Chen et al., 2016). We further compared the ASD/NC classification accuracy of our method with two novel classification frameworks that combine both supervised and unsupervised techniques: (1) SIMLR-based pairing + SVM, and (2) HWC-based pairing + SVM. Each of these methods was evaluated on (i) the concatenated low-order morphological brain networks (i.e., four views) (CON), and (ii) the high-order morphological brain network (HON) for both left and right hemispheres.

### 3.3. The most discriminative features for ASD diagnosis

Based on the results of our proposed clustering framework, we identified the most discriminative low-order and high-order morphological connectional biomarkers that discriminate between ASD and NC subjects. Specifically, to rank each morphological connectional feature **f**, we adopt the Laplacian score:

(2) $$\begin{array}{c} LS\left(f\right)=\frac{f^{\prime }Sf}{f^{\prime }f} \end{array}$$

LS quantifies the concordance between the features and the similarity (He et al., 2005).

**Figure 3:**
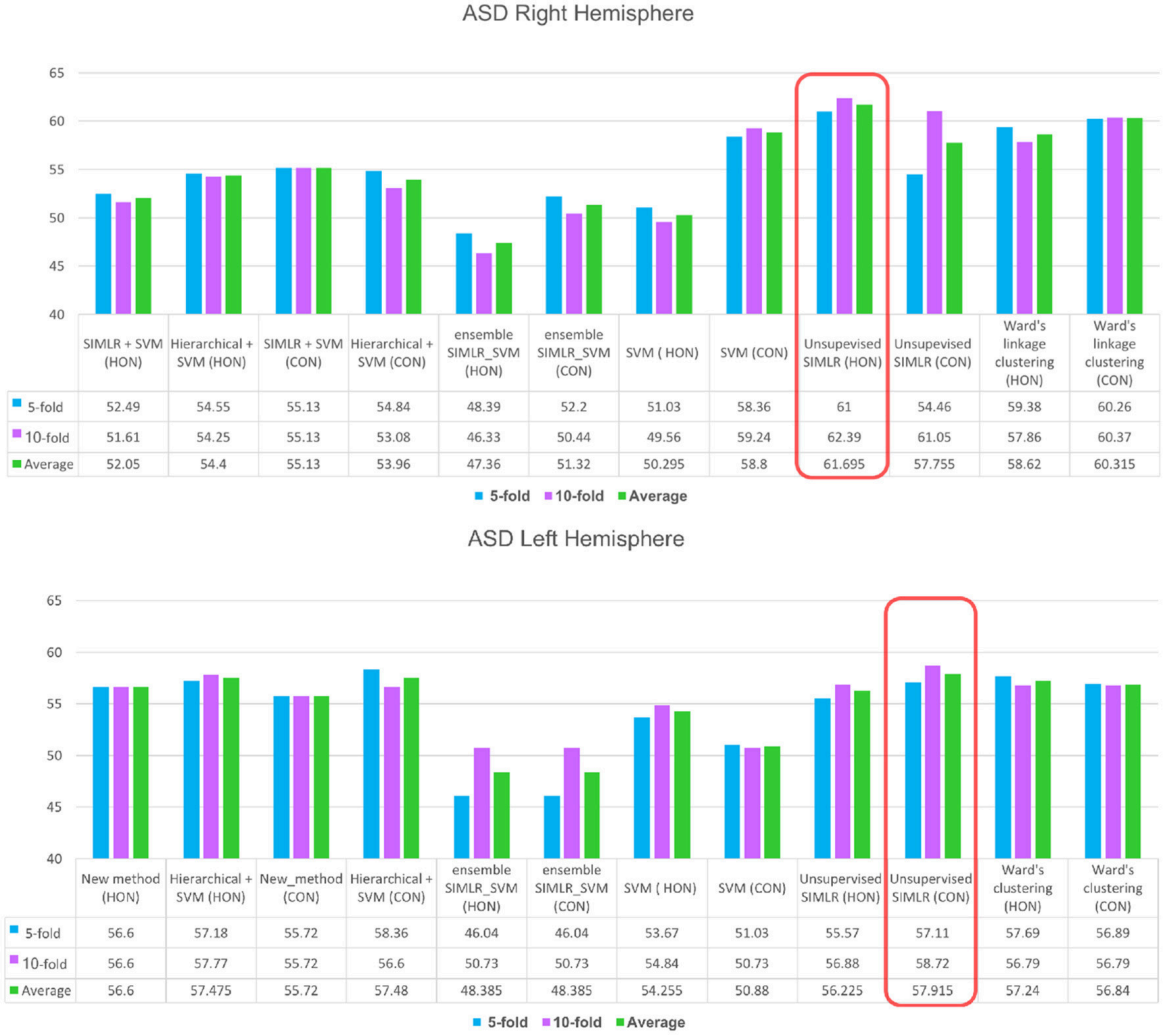
ASD identification accuracy using our method and comparison supervised and unsupervised methods. We evaluated each of these methods on (i) the concatenated low-order morphological networks (i.e., 4 views) that we term with CON, and (ii) the high-order morphological networks (HON).

For the right hemisphere (RH), our method (unsupervised SIMLR HON) had the best performance in distinguishing between ASD/NC subjects among all methods using both 5-fold and 10-fold cross validation schemes with an average performance of 61.7% (Figure 3), which might indicate that the RH features have more discriminative power at a higher order level. We report that an increase in accuracy was also observed with the (HWC-based pairing + SVM) using HON features. However, for all other methods, a higher accuracy was obtained using morphological CON features. This might reflect the large heterogeneity of ASD disorder in the way it affects *morphological brain networks* and its unpredictable behavior across different classifiers. Previous studies pointed to the large heterogeneity present in autistic subjects (Lenroot and Yeung, 2013; Masi et al., 2017) and how can this lead sometimes to conflicting results in terms of identified biomarkers (Orekhova and Stroganova, 2014).

As for the left hemisphere (LH), unsupervised SIMLR CON achieved the best mean average accuracy across all methods (Figure 3), which might indicate that morphological connections between LH regions altered by ASD occur at a low-order level. In other words, the LH pairwise connectivity weight between regions in the same morphological view depicts better the changes associated with autism than the high-order relationship between pairs of regions across different views. Although our proposed framework scored better with the low-order network, we notice that all comparison methods produced slightly better results when using high-order networks.

**Figure 4:**
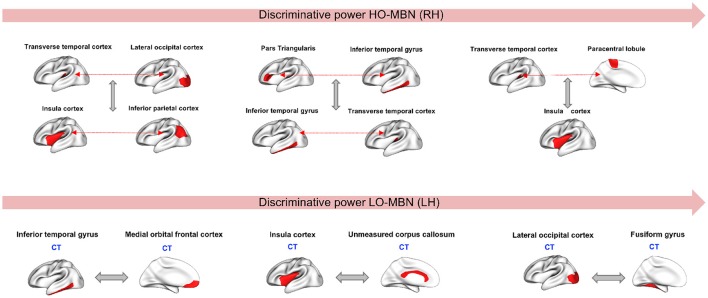
Top three discriminative high-order and low-order features in right and left hemispheres, respectively. CT, Cortical thickness view.

Since our aim is to find the most discriminative morphological connections, we identified the top three features by our method achieving the best classification accuracies for both hemispheres across all 10 and 5-fold cross-validation runs that showed consistency in results. Using the Laplacian score, the most discriminative high-order morphological connectional features for the right hemisphere connecting two pairs of ROIs are: (1) (transverse temporal cortex, paracentral lobule) with insula cortex (2) (inferior temporal gyrus, Pars triangular) and (transverse temporal cortex, inferior temporal gyrus), (3) (transverse temporal cortex, lateral occipital cortex) with (insula cortex, inferior parietal cortex). For the left hemisphere, the top low-order connectional features connecting two ROIs are: (1) lateral occipital cortex and fusiform gyrus, (2) insula cortex and unmeasured corpus collosum, (3) inferior temporal gyrus and medial orbital frontal cortex (Figure 4). We also notice that the identified regions at a high-order level are different from those at a lower-order, which can provide complementary discriminative information for more accurate diagnosis.

## 4. Discussion

In this paper, we introduced a different type of high-order brain network, that explores brain regional relationships beyond the physical brain connectivity derived from structural networks and statistical dependencies obtained from functional networks (Bullmore and Sporns, 2009). In particular, our high-order brain network investigates *shape-to-shape ‘connections’* among pairs of brain regions. We aimed to identify morphological connectional biomarkers for distinguishing between autistic and healthy subjects. Specifically, we proposed two types of morphological brain network representations: the low-order morphological brain network, which captures the relationship in cortical morphology between only two brain regions, and (2) the high-order morphological brain network which explores the relationship between multiple brain regions.

Our proposed frameworks using SIMLR on both CON and HON achieved better performance than baseline methods for LH and RH. This shows the ability of unsupervised SIMLR to disentangle heterogeneous patterns related to autism disorder compared to other methods. This might be explained by the use of multiple kernels for connectomic manifold learning, which enables to capture a wide spectrum of inherent statistical distributions (from flat to compact) of ASD/NC subjects. We should note also that despite the accurate results reported in previous studies about ensemble classifiers (Lima et al., 2003; Džeroski and Ženko, 2004; Quan et al., 2016), our proposed unsupervised framework scored better when using average cross-validation than ensemble SVMs (HWC-based pairing + SVM and SIMLR-based pairing + SVM). Their low performance can be explained by the fact that SIMLR and HWC tend to produce more homogeneous clusters, hence creating a non-balanced data samples in the pairing stage for SVM training. This points to the imbalanced data issue for training supervised methods that is difficult to alleviate using ensemble classifiers.

Through using the low-order and high-order networks and identifying the most discriminative features, we found that cortical thickness has the highest discriminative power among the four used cortical attributes (Figure 4). Several studies have reported cortical thickness as an important morphological biomarker for ASD (M.K.Chung et al., 2005; Hardan et al., 2006; Zielinski et al., 2014; Smith et al., 2016; Khundrakpam et al., 2017) where they showed that abnormalities caused by autism is coupled with an increase of cortical thickness in ASD subjects compared to healthy controls. Similar findings were reported in Courchesne et al. (2001) and Hazlett et al. (2005) where they used a volume-based imaging to report an abnormal enlargement in total brain volume among very young children (age between 18 month and 4 years). The most discriminative morphological brain connections with the highest Laplacian score were found at both low- and high-order connectional levels. These mainly included the insula cortex (IC) and lateral occipital cortex (LOC), which were most frequently selected. It is known that IC is related to cognitive, affective and sensorimotor processing. Yamada et al. (2016) showed that IC displayed structural and functional abnormalities in ASD. Many other studies have reported the correlation between IC dysfunctionality and autism when it comes to tasks related to emotional and social processing (Uddin and Menon, 2009; Caria and de Falco, 2015). In addition, LOC plays an important role in human object recognition (Grill-Spector et al., 2001). Dawson et al. (2002) conducted a study on autistic subjects vs. typically developing children and found that children with ASD failed to show differences in their high-density brain event-related potentials (ERP) to a familiar vs. an unfamiliar face but they were able to show differences when it comes to processing a familiar vs. an unfamiliar object. Another study (Kuusikko-Gauffin et al., 2011) confirmed these findings where it showed that autism is related to impairments in face memory and face recognition but intact object recognition. On the other hand, the fusiform gyrus is involved in the processing of face and body recognition (Furl et al., 2011) which explains its connection to LOC. We also found that other identified regions in our work such as unmeasured corpus collosum (UCC) and medial orbital frontal cortex (MOFC), involved in emotional and cognitive processing, learning and social behavior, were largely investigated and had abnormal patterns in ASD (Hardan et al., 2006; Girgis et al., 2007; He et al., 2010; Prigge et al., 2013; Wolff et al., 2015). We can conclude from all these findings that our identified ‘morphological’ regions are in agreement with the behavioral phenotype of ASD derived from other data types (e.g., functional MRI).

Our study has few limitations. First, on a low-order level, despite using different types of morphological attributes, we used a simple concatenation of all views to extract the features. Second, on a high-order level, we used Pearson correlation to explore the connections between multiple regions which may overlook the non-linear relationship between them. Third, although we identified morphological connectional biomarkers for ASD identification, we did not investigate the connection between the discovered cortical regions to non-cortical regions. Fourth, in addition to investigating how ASD alters the relationship between brain morphologies using multi-view brain networks, we expect that by integrating structural and functional networks into our framework, we could provide a more holistic connectomic understanding of how ASD affects the different connectional facets of the brain construct, which might result in further improving the classification performance. Last, we did not use any feature selection methods to further enhance the performance of our framework. These unexplored directions can be investigated in our future work.

## 5. Conclusion

In this paper, we presented the first work on a high-order connectomic manifold learning using morphological brain networks for autism identification. Our framework outperformed both supervised and unsupervised baseline methods as well as a set of ensemble learning frameworks and was able to further identify the most discriminative relationships between *pairs of morphological brain connections*. Noting that ASD classification is a challenging problem, achieving 61.69% is quite promising based on solely T1-w MR images. To improve the connectomic manifold learning for a more accurate ASD/NC segregation, we will leverage multi-view feature selection methods such as Liu et al. (2015). Since our unsupervised learning method is generic, we can also use it to investigate other neurological disorders such as dementia.

## Author contributions

All authors listed have made a substantial, direct and intellectual contribution to the work, and approved it for publication.

### Conflict of interest statement

The authors declare that the research was conducted in the absence of any commercial or financial relationships that could be construed as a potential conflict of interest.

## Footnotes

^1^http://fcon_1000.projects.nitrc.org/indi/abide/

## References

[B1] Abdi H. Williams L. J. (2010). Principal Component Analysis. Wiley Interdisc Rev. 2, 433–459. 10.1002/wics.101

[B2] Anderson J. S. Nielsen J. A. Froehlich A. L. DuBray M. B. Druzgal T. J. Cariello A. N.. (2011). Functional connectivity magnetic resonance imaging classification of autism. Brain 134, 3742–3754. 10.1093/brain/awr263 22006979 PMC3235557

[B3] Brown C. Hamarneh G. (2016). Machine learning on human connectome data from MRI. arXiv:1611.08699v1.

[B4] Buckner R. L. Krienen F. M. Yeo B. T. (2013). Opportunities and limitations of intrinsic functional connectivity mri. Nat. Neurosci. 7, 832–837. 10.1038/nn.3423 23799476

[B6] Bullmore E. Sporns O. (2009). Complex brain networks: graph theoretical analysis of structural and functional systems. Nat. Neurosci. 10, 186–198. 10.1038/nrn2575 19190637

[B5] Bullmore E. T. Bassett D. S. (2011). Brain graphs:graphical models of the humain brain connectome. Annu. Rev. Clin. Psychol. 7, 113–140. 10.1146/annurev-clinpsy-040510-143934 21128784

[B7] Caria A. de Falco S. (2015). Anterior insular cortex regulation in autism spectrum disorders. Front. Behav. Neurosci. 9:38. 10.3389/fnbeh.2015.00038 25798096 PMC4351628

[B8] Chen H. Iraji A. Jiang X. Lv J. Kou Z. Liu T. (2015). Longitudinal analysis of brain recovery after mild traumatic brain injury based on groupwiseconsistent brain network clusters. Springer 9350, 194–201. 10.1007/978-3-319-24571-3_24

[B9] Chen X. Zhang H. Shen D. (2016). Ensemble hierarchical high-order functional connectivity networks for MCI classification, in International Conference on Medical Image Computing and Computer-Assisted Intervention (Athens: MICCAI), 18–25. 10.1007/978-3-319-46723-8_3 PMC5604247 28936492

[B10] Courchesne E. Karns C. M. Davis H. R. Ziccardi R. Carper R. A. Tigue Z. D.. (2001). Unusual brain growth patterns in early life in patients with autistic disorder. Neurology 57, 245–254. 10.1212/WNL.57.2.245 11468308

[B11] Dawson G. Carver L. Meltzoff A. N. Panagiotides H. McPartland J. Webb S. J. (2002). Neural correlates of face and object recognition in young children with autism spectrum disorder, developmental delay, and typical development. Child Develop. 73, 700–717. 10.1111/1467-8624.00433 12038546 PMC3651041

[B12] Dietterich T. G. (2000). Ensemble methods in machine learning. Multiple Classif. Sys. 1857, 1–15. 10.1007/3-540-45014-9_1

[B13] Džeroski S. Ženko B. (2004). Is combining classifiers with stacking better than selecting the best one? Mach. Learn. 54, 255–273. 10.1023/B:MACH.0000015881.36452.6e

[B14] Ecker C. Marquand A. Mourão-Miranda J. Johnston P. Daly E. M Brammer M. J. (2010). Describing the brain in autism in five dimensions–magnetic resonance imaging-assisted diagnosis of autism spectrum disorder using a multiparameter classification approach. J. Neurosci. 32, 10612–10623. 10.1523/JNEUROSCI.5413-09.2010 PMC6634684 20702694

[B15] Ecker C. Rocha-Rego V. Johnston P. Mourao-Miranda J. Marquand A. Daly E. M.. (2009). Investigating the predictive value of whole-brain structural mr scans in autism: A pattern classification approach. Neuroimage 49, 44–56. 10.1016/j.neuroimage.2009.08.024 19683584

[B16] Fischl B. (2012). Freesurfer. Neuroimage 62, 774–781. 10.1016/j.neuroimage.2012.01.021 22248573 PMC3685476

[B17] Furl N. Garrido L. Dolan R. J. Driver J. Duchaine B. (2011). Fusiform gyrus face selectivity relates to individual differences in facial recognition ability. J. Cogn. Neurosci. 23, 1723–1740. 10.1162/jocn.2010.21545 20617881 PMC3322334

[B18] Gao H. Cai C. Yan J. Yan L. Cortes J. G. Wang Y.. (2015). Identifying connectome module patterns via new balanced multi-graph normalized cut. Springer 9350, 169–176. 10.1007/978-3-319-24571-3_21 26525952 PMC4624338

[B19] Girgis R. R. Minshew N. J. Melhem N. M. Nutche J. J. Keshavan M. S. Hardan A. Y. (2007). Volumetric alterations of the orbitofrontal cortex in autism. Progr. Neuro Psychopharmacol. Biological Psychiatry 31, 41–45. 10.1016/j.pnpbp.2006.06.007 16863674 PMC2888006

[B20] Grill-Spector K. Kourtzi Z. Kanwisher N. (2001). The lateral occipital complex and its role in object recognition. Vis. Res. 41, 1409–1422. 10.1016/S0042-6989(01)00073-6 11322983

[B21] Hardan A. Muddasani S. Vemulapalli M. Keshavan M. Minshew N. (2006). An mri study of increased cortical thickness in autism. Am. J. Psychiatry 163, 1290–1292. 10.1176/ajp.2006.163.7.1290 16816240 PMC1509104

[B22] Hazlett H. C. Poe M. Gerig G. Smith R. G. Provenzale J. Ross A.. (2005). Magnetic resonance imaging and head circumference study of brain size in autism: birth through age 2 years. Arch. Gen. Psychiatry 62, 1366–1376. 10.1001/archpsyc.62.12.1366 16330725

[B23] He Q. Duan Y. Karsch K. Miles J. (2010). Detecting corpus callosum abnormalities in autism based on anatomical landmarks. Psychiatry Res. Neuroimaging 183, 126–132. 10.1016/j.pscychresns.2010.05.006 20620032 PMC2910223

[B24] He X. Cai D. Niyogi P. (2005). Laplacian score for feature selection, in Proceedings of the 18th International Conference on Neural Information Processing Systems (NIPS'05), eds Weiss Y. Schökopf B. Platt J. C. (Cambridge, MA: MIT Press) 507–514.

[B25] Ingalhalikar M. Kanterakis S. Gur R. Roberts T. Verma R. (2010). Dti based diagnostic prediction of a disease via pattern classification. Med. Image Comput. Comput. Assist. Interv. 2, 558–565. 10.1007/978-3-642-15705-9_68 20879275

[B26] Jbabdi S. Johansen-Berg H. (2011). Tractography: where do we go from here? Brain Connect 1, 169–183. 10.1089/brain.2011.0033 22433046 PMC3677805

[B27] Joe H. Ward J. (1963). Hierarchical grouping to optimize an objective function. J. Am. Statist. Assoc. 58, 236–244. 10.1080/01621459.1963.10500845

[B28] Khundrakpam B. Lewis J. Kostopoulos P. Carbonell F. Evans A. (2017). Cortical thickness abnormalities in autism spectrum disorders through late childhood, adolescence, and adulthood: A large-scale mri study. Cereb. Cortex 27, 1721–1731. 10.1093/cercor/bhx038 28334080

[B29] Kuusikko-Gauffin S. Eira J.-V. Alice C. Rachel P.-W. Katja J. Marja-Leena M. (2011). Face memory and object recognition in children with high-functioning autism or asperger syndrome and in their parents. Res. Autism Spectr. Disord. 5, 622–628. 10.1016/j.rasd.2010.07.007

[B30] Landa R. J. (2008). Diagnosis of autism spectrum disorders in the first 3 years of life. Nat. Rev. Neurol. 4, 138–147. 10.1038/ncpneuro0731 18253102

[B31] Lanyon L. (2012). Diffusion Tensor Imaging: Structural Connectivity Insights, Limitations and Future Directions. Casablanca: INTECH.

[B32] Lenroot R. K. Yeung P. K. (2013). Heterogeneity within autism spectrum disorders: What have we learned from neuroimaging studies? Front. Hum. Neurosci. 7:733. 10.3389/fnhum.2013.00733 24198778 PMC3812662

[B33] Lima C. Coelho A. ZUBEN F. V. (2003). Ensembles of support vector machines for classification tasks with reduced training sets. WSEAS Trans. Sys. 2, 370–375.

[B34] Lisowska A. Rekik I. (2018). Joint pairing and structured mapping of convolutional brain morphological multiplexes for early dementia diagnosis. Brain connect. 10.1089/brain.2018.0578 29926746 PMC6909728

[B35] Lisowska A. Rekik I. Initiative A. D. N. (2017). Pairing-based ensemble classifier learning using convolutional brain multiplexes and multi-view brain networks for early dementia diagnosis, in International Workshop on Connectomics in Neuroimaging (Cham) 42–50. 10.1007/978-3-319-67159-8_6

[B36] Liu Y. Liao B. Han Y. (2015). Discriminative multi-view feature selection and fusion, in Multimedia and Expo (ICME), 2015 IEEE International Conference, 1–6. 10.1109/ICME.2015.7177432

[B37] Lord C. Cook E. H. Leventhal B. L. Amaral D. G. (2000). Autism spectrum disorders. Neuron 28, 355–363. 10.1016/S0896-6273(00)00115-X 11144346

[B38] M.K.Chung M. Robbins S. M.Dalton K. J.Davidson R. L.Alexander A. C. Evans A. (2005). Cortical thickness analysis in autism with heat kernel smoothing. Neuroimage 25, 1256–1265. 10.1016/j.neuroimage.2004.12.052 15850743

[B39] Maaten L. Hinton G. (2008). Visualizing data using t-sne. Jo. Mach. Learn. Res. 9, 2579–2605.

[B40] Mahjoub I. Mahjoub M. A. Rekik I. (2018). Brain multiplexes reveal morphological connectional biomarkers fingerprinting late brain dementia states. Sci. Reports 8:4103. 10.1038/s41598-018-21568-7 29515158 PMC5841319

[B41] Masi A. DeMayo M. M. Glozier N. Guastella A. J. (2017). An overview of autism spectrum disorder, heterogeneity and treatment options. Neurosci. Bullet. 33, 183–193. 10.1007/s12264-017-0100-y 28213805 PMC5360849

[B42] Mueller S. G. Weiner M. W. Thal L. J. Petersen R. C. Jack C. Jagust W. (2005). The Alzheimer's disease neuroimaging initiative. Neuroimaging Clin N.Am. 10, 869–877. 10.1016/j.nic.2005.09.008 PMC2376747 16443497

[B43] Nadler B. Galun M. (2006). Fundamental limitations of spectral clustering, in Advances in neural information processing systems, eds Schölkopf B. Platt J. C. Hoffman T. (MIT Press), 1017–1024.

[B44] Nielsen J. A. Zielinski B. A. Fletcher P. T. Alexander A. L. Lange N. Bigler E. D.. (2013). Multisite functional connectivity mri classification of autism: abide results. Front. Hum. Neurosci. 7:599. 10.3389/fnhum.2013.00599 24093016 PMC3782703

[B45] Orekhova E. V. Stroganova T. A. (2014). Arousal and attention re-orienting in autism spectrum disorders: evidence from auditory event-related potentials. Front. Hum. Neurosci. 8:34. 10.3389/fnhum.2014.00034 24567709 PMC3915101

[B46] Petrov D. Ivanov A. Faskowitz J. Gutman B. Moyer D. Villalon J. (2017). Evaluating 35 methods to generate structural connectomes using pairwise classification. arXiv:1706.06031 10.1007/978-3-319-66182-7_59

[B47] Pillai A. S. McAuliffe D. Lakshmanan B. M. Mostofsky S. H. Crone N. E. Ewen J. B. (2018). Altered task-related modulation of long-range connectivity in children with autism. Autism Res. 11, 245–257. 10.1002/aur.1858 28898569 PMC5825245

[B48] Pollonini L. Patidar U. Situ N. Rezaie R. Papanicolaou A. C. Zouridakis G. (2010). Functional connectivity networks in the autistic and healthy brain assessed using granger causality, in Annual International Conference of the IEEE EMBS (Buenos Aires), 1730–1733. 10.1109/IEMBS.2010.5626702 21096408

[B49] Price T. Wee C. Y. Gao W. Shen D. (2014). Multiple-network classification of childhood autism using functional connectivity dynamics. Med. Image Comput. Comput. Assist. Interv. 17, 177–184. 10.1007/978-3-319-10443-0_23 25320797

[B50] Prigge M. B. Lange N. Bigler E. D. Merkley T. L. Neeley E. S. Abildskov T. J.. (2013). Corpus callosum area in children and adults with autism. Res. Autism Spectrum Disord. 7, 221–234. 10.1016/j.rasd.2012.09.007 23130086 PMC3487714

[B51] Quan Y. Xu Y. Sun Y. Huang Y. Ji H. (2016). Sparse coding for classification via discrimination ensemble, in Proceedings of the IEEE Conference on Computer Vision and Pattern Recognition, 5839–5847. 10.1109/CVPR.2016.629

[B52] Rudie J. D. Brown J. A. Beck-Pancer D. Hernandez L. M. Dennis E. L. Thompson P. M.. (2013). Altered functional and structural brain network organization in autism. Neuroimage 2, 79–94. 10.1016/j.nicl.2012.11.006 24179761 PMC3777708

[B53] Sahyoun C. P. Belliveau J. W. Soulières I. Schwartz S. Mody M. (2010). Neuroimaging of the functional and structural networks underlying visuospatial vs. linguistic reasoning in high-functioning autism. Neuropsychologia 48, 86–95. 10.1016/j.neuropsychologia.2009.08.013 19698726 PMC2795068

[B54] Sato J. R. Balardin J. Vidal M. C. Fujita A. (2016). Identification of segregated regions in the functional brain connectome of autistic patients by a combination of fuzzy spectral clustering and entropy analysis. J. Pscychiatry Neurosci. 2, 124–132. 10.1503/jpn.140364 PMC4764481 26505141

[B55] Smith E. Thurm A. Greenstein D. Farmer C. Swedo S. Giedd J.. (2016). Cortical thickness change in autism during early childhood. Hum. Brain Mapp. 37, 2616–2229. 10.1002/hbm.23195 27061356 PMC4905793

[B56] Soussia M. Rekik I. (2017). High-order connectomic manifold learning for autistic brain state identification, in Proceedings of Connectomics in NeuroImaging: First International Workshop, CNI 2017, Held in Conjunction with MICCAI 2017, Lecture Notes in Computer Science, Vol. 10511 (Quebec City, QC), 51–59.

[B57] Sparks B. F. Friedman S. D. Shaw D. W. Aylward E. H. Echelard D. Artru A. A.. (2002). Brain structural abnormalities in young children with autism spectrum disorder. Neurology 59, 184–192. 10.1212/WNL.59.2.184 12136055

[B58] Stigler K. A. McDonald B. C. Anand A. Saykin A. J. McDougle C. J. (2011). Structural and functional magnetic resonance imaging of autism spectrum disorders. Neuropsychologia 1380, 146–161. 10.1016/j.brainres.2010.11.076 21130750 PMC3465665

[B59] Tsiaras V. Simos P. Rezaie R. Sheth B. Garyfallidis E. Castillo E.. (2011). Extracting biomarkers of autism from meg resting-state functional connectivity networks. Comput. Biol. Med. 41, 1166–1177. 10.1016/j.compbiomed.2011.04.004 21592470

[B60] Uddin L. Q. Menon V. (2009). The anterior insula in autism: under-connected and under-examined. Neurosci. Biobehav. Rev. 33, 1198–1203. 10.1016/j.neubiorev.2009.06.002 19538989 PMC2743776

[B61] Wang B. Zhu J. Pierson E. Ramazzotti D. Batzoglou S. (2017). Visualization and analysis of single-cell rna-seq data by kernel-based similarity learning. Nature 70, 869–879. 10.1101/052225 28263960

[B62] Wang X. Sontag D. Wang F. (2014). Unsupervised learning of disease progression models, in KDD'14 Proceedings, 85–94.

[B63] Wolff J. J. Gerig G. Lewis J. D. Soda T. Styner M. A. Vachet C.. (2015). Altered corpus callosum morphology associated with autism over the first 2 years of life. Brain 138, 2046–2058. 10.1093/brain/awv118 25937563 PMC4492413

[B64] Yamada T. Itahashi T. Nakamura M. Watanabe H. Kuroda M. Ohta H.. (2016). Altered functional organization within the insular cortex in adult males with high-functioning autism spectrum disorder: evidence from connectivity-based parcellation. Mol. Autism 7:41. 10.1186/s13229-016-0106-8 27713815 PMC5052801

[B65] Yang J. Leskovec J. (2010). Modeling information diffusion in implicit networks, in Data Mining (ICDM), 2010 IEEE 10th International Conference, 599–608. 10.1109/ICDM.2010.22

[B66] Zhao F. Zhang H. Rekik I. Shen D.. (2018). Diagnosis of Autism Spectrum Disorders using multi-level high-order functional networks derived from resting-state functional MRI. Front. Hum. Neurosci. 12:184. 10.3389/fnhum.2018.00184 29867410 PMC5960713

[B67] Zhou Y. Qiao L. Li W. Zhang L. Shen D. (2018). Simultaneous estimation of low-and high-order functional connectivity for identifying mild cognitive impairment. Front. Neuroinform. 12:3. 10.3389/fninf.2018.00003 29467643 PMC5808180

[B68] Zhu Y. Zhu X. Zhang H. Gao W. Shen D. Wu G. (2016). Reveal consistent spatial-temporal patterns from dynamic functional connectivity for autism spectrum disorder identification, in International Conference on Medical Image Computing and Computer-Assisted Intervention (Quebec: Springer, Cham), 106–114. 10.1007/978-3-319-46720-7_13 PMC5278798 28149963

[B69] Zielinski B. A. Prigge M. B. Nielsen J. A. Froehlich A. L. Abildskov T. J. Anderson J. S (2014). Longitudinal changes in cortical thickness in autism and typical development. Brain 136, 1799–1812. 10.1093/brain/awu083 PMC4032101 24755274

